# Oleander Stem and Root Standardized Extracts Mitigate Acute Hyperglycaemia by Limiting Systemic Oxidative Stress Response in Diabetic Mice

**DOI:** 10.1155/2019/7865359

**Published:** 2019-01-08

**Authors:** Priyankar Dey, Manas Ranjan Saha, Sumedha Roy Choudhuri, Indrani Sarkar, Biswajit Halder, Mousumi Poddar-Sarkar, Arnab Sen, Tapas Kumar Chaudhuri

**Affiliations:** ^1^Cellular Immunology Laboratory, Department of Zoology, University of North Bengal, Siliguri, West Bengal 734013, India; ^2^Human Nutrition Program, Department of Human Sciences, The Ohio State University, Columbus, Ohio 43210, USA; ^3^Molecular Cytogenetics Laboratory, Department of Botany, University of North Bengal, Siliguri, West Bengal 734013, India; ^4^Chemical Signal and Lipidomics Laboratory, Department of Botany, University of Calcutta, Kolkata, West Bengal 700019, India; ^5^Department of Pathology, North Bengal Medical College, West Bengal 734011, India; ^6^Visiting Professor, Department of Zoology, Bodoland University, Kokrajhar, Assam 734011, India

## Abstract

The extracts of different parts of *Nerium oleander* L. are used as antidiabetic remedy in the traditional medicinal systems of different parts of the world. Despite these uses in ethnomedicinal system, the antihyperglycemic potentials of oleander stem (NOSE) and root (NORE) extracts have not been pharmacologically evaluated. Therefore, we aimed at evaluating the antidiabetic ethnomedicinal claims of NOSE and NORE, primarily focusing on glucose homeostasis and associated metabolic implications. Alloxan-treated mice with hyperglycaemia (blood glucose >200 mg/dL) were treated with oleander 70% hydromethanolic extracts (200 mg/kg) for 20 consecutive days, and the results were compared with positive control glibenclamide. Blood glucose level was 52–65% lowered (*P* < 0.001) in oleander treated groups, which was otherwise 4.62 times higher in diabetic mice, compared to control. Insulin resistance was lowered 51–36% irrespective of any significant (*P* > 0.05) changes in insulin sensitivity throughout the treatments. Improved serum insulin remained associated with lowered glucose level (*r*_P_ = −0.847 and −0.772; *P* < 0.01). Markers of hyperglycaemia-related hepatic glycogen, glycated haemoglobin (HbA1c), hyperlipidaemia, hepatic injury, and diabetic nephropathy were normalized as well. Improvement of systemic intrinsic antioxidant enzymes (catalase and peroxidase) were correlated (*r*_P_ = −0.952 to −0.773; *P* < 0.01) with lower lipid peroxidation by-product malondialdehyde (MDA) in the circulation. Principal component analysis coupled with hierarchical cluster analysis represented shift in metabolic homeostasis in diabetic mice, which was further normalized by oleander and glibenclamide treatment. Additionally, molecular docking studies of the phenolic acids measured by HPLC with intracellular cytoprotective transcription factor nuclear factor erythroid 2-related factor 2 (Nrf2) revealed strong molecular interactions. The results collectively support the ethnomedicine antidiabetic claims of oleander stem and root and suggest that the oleander mediated elevation of systemic antioxidant status is likely responsible for the improved glycaemic control.

## 1. Introduction

Diabetes mellitus (DM) is a systemic disorder, primarily characterized by loss of glucose homeostasis, impaired insulin signalling, carbohydrate, and lipid metabolism, ultimately resulting in progressive systemic complications such as hyperlipidemia, hyperglycaemia, nephropathy, hepatic injury, vascular dysfunction, etc. The global prevalence of diabetes has quadrupled in the last 30 years [1]. It is expected to deteriorate based on estimate that 642 million people will be diabetic, and an additional 481 million will suffer from impaired glucose tolerance by the end of 2040 [2].

*Nerium oleander* L. (syn. *Nerium indicum* Mill and *Nerium odorum* Aiton) is an ethnomedicinal shrub which is used for the treatment of diabetic complications in India, Pakistan, Algeria, and Morocco, and its medicinal properties are recognized in Ayurveda as well [3]. Prior pharmacognostic studies have demonstrated that oleander at 200 mg/kg/day lowers hyperinsulinemia and hyperglycaemia in association with attenuating streptozotocin-induced diabetic hepatotoxicity in rats [4]. Methanolic exact of oleander leaf also ameliorates alloxan-induced diabetes by improving antioxidant enzymes and limiting lipid peroxidation in mice [5]. These benefits over glycaemic control were irrespective of type of extracts as prior reports suggest that several fractions of oleander leaf limits hyperglycaemia in rats [6].

However, despite its tremendous traditional therapeutic use, pharmacognostic studies are focused primarily only on the bioactivities of its leaves. Interestingly, recent prospective studies have demonstrated potent free-radical scavenging properties and comparable bioactivities of oleander stem and root extracts to modulate macrophage bioactivities and limit haloalkane-induced hepatotoxicity in murine models [7–9]. These reports were additionally associated with detailed phytochemical fingerprinting using chromatographic and spectroscopic techniques that revealed several bioactive constituents in different parts of oleander.

Therefore, based on the ethnomedicinal use against diabetic complications and pharmacognostic reports of hepatoprotective activities, we hypothesized that oleander stem and root hold the potentiality to lower hyperglycaemia and improve glucose homeostasis and insulin resistance by improving systemic oxidative stress against a free-radical induced murine model of type 1 diabetes (T1D). The extracts were further analysed by HPLC, and *in silico* docking studies were performed against the intrinsic cytoprotective transcription factor to validate the hypothesis.

## 2. Materials and Methods

### 2.1. Chemicals and Solvents

All chemicals were obtained from HiMedia Laboratories Pvt. Ltd. (Mumbai, India), unless otherwise indicated. Spectrophotometric kits to measure serum chemistries were procured from Crest Biosystem; Goa, India. HPLC standards were purchased from Sigma-Aldrich (St. Louis, MO, USA) and ChromaDex (Irvine, CA, USA). MilliQ water (Ω 18.2) from departmental central facility was used for all the experiments.

### 2.2. Animals

Inbred Swiss albino mice (7-8 wk; male) were maintained (25 ± 2°C; 12 h photoperiod) in the departmental animal house, with food and water *ad libitu*m. Female mice were excluded from the study based on the report that female mice are resistant to experimental obesity and insulin resistance [10]. All *in vivo* experiments were reviewed and approved by the university ethical committee (840/ac/04/CPCSEA).

### 2.3. Plant Sample and Extract Preparation

Stem and root of white flowered variety of oleander were collected from departmental garden (26.71°N, 88.35°S) during August 2015. Plant samples were authenticated, and voucher specimen was stored at the university herbarium with an accession no. 09618. Hydromethanolic (70%) extracts of stem (NOSE) and root (NORE) were prepared as described previously [8]. In brief, shade-dried and grinded 100 g of oleander stem and root were separately mixed with 1000 mL of 70% methanol and twice extracted for 12 h at 37°C. Extraction was performed under reduced pressure and at physiological temperature to preserve the heat labile phytochemicals. The resultant was filtered and concentrated under reduced pressure and lyophilized. The final yield of NOSE and NORE were 11.82% and 15.22% of dry weight.

### 2.4. Experimental Design

Freshly prepared alloxan monohydrate (in 154 mM NaCl) was intraperitoneally injected (200 *µ*L; 150 mg/kg) to each mouse. Mouse with blood glucose level >200 mg/dL post 3 d of injection was considered diabetic and randomized (body weight (BW); *P* > 0.05) into following cohorts (*n*=6): *T1D* group receive normal saline; *Glib* group received glibenclamide (5 mg/kg/d) as reference drug; *NOSE* and *NORE* groups were gavaged 200 mg/kg/d NOSE and NORE, respectively. Another cohort of 6 nondiabetic mice (not alloxanized; glucose <60 mg/dL) were considered as control (Ctrl). Doses of NOSE and NORE were selected based on prior reports of acute toxicity study which demonstrated that up to 2 g/kg BW oral gavaging of the extracts display no clinical and toxicological symptoms in mice, in addition to ameliorate haloalkane-induced hepatotoxicity at 200 mg/BW dose [9]. Based on these evidences, 200 mg/BW dose was selected for the present study which was 1/10^th^ of the highest dose (2 g/kg) selected in acute toxicity study.

Blood glucose level was measured from the tail vein using one-touch glucometer (Bayer, contour TS meter), and BW was monitored periodically. After 20 d, mice were fasted 12 h and whole blood was collected from the heart under mild ether anaesthesia, allowed to colt for 30 min and centrifuged (2000 × g; 15 min) to obtain serum. Mice were sacrificed by cervical dislocation, and liver, both kidneys, and thigh skeletal muscle were collected, washed in phosphate buffer saline, snap-frozen, and stored in −80°C freeze until further use.

### 2.5. Measurement of Hyperglycaemic Metabolic Parameters

Serum insulin was estimated by the ELISA method using Accu-Bind Universal ELISA kit (Monobind Inc., USA) according to manufacturer's instructions, and glycated haemoglobin (HbA1c) level was measured by ion-exchange HPLC using D-10™ Dual HbA1c program (Bio-Rad #220-0201). Insulin resistance was calculated using the Homeostatic Model Assessment of Insulin Resistance (HOMA-IR): glucose (mg/dL) × insulin (mU/L). Insulin sensitivity was calculated using the Quantitative Insulin Sensitivity Check Index (QUICKI): 1/log (fasting insulin (mU/mL) + log (fasting glucose (mg/dL)). Hepatic glycogen content was measured according to a standardized Anthrone reagent method [11] against a glucose standard curve prepared in parallel to the samples.

### 2.6. Measurement of Serum Chemistries

Serum biochemical parameters (acid phosphatase (ACP); alanine aminotransferase (ALP); alanine transaminase (ALT); aspartate transaminase (AST); blood urea nitrogen (BUN); cholesterol, triglyceride, creatinine, and uric acid) were spectrophotometrically measured as primary markers of diabetic hepatic injury, hyperlipidemia, and diabetic nephropathy, using commercially available kits (Crest Biosystem; Goa, India).

### 2.7. Measurement of Antioxidant Enzymes and Lipid Peroxidation

Catalase (CAT) activity was studied by measuring breakdown of H_2_O_2_ at 240 nm [12], and peroxidase (PX) activity was measuring the oxidation of guiacol at 436 nm according to standardized methods [13]. The extent of lipid peroxidation was measured in terms of serum malondialdehyde (MDA) content by colorimetric thiobarbituric acid reactive substances (TBARS) assay kit (Cayman Chemical, USA) as per the manufacturer's instructions.

### 2.8. Oral Glucose Tolerance Test (OGTT)

In brief, a separate cohort of alloxanized (glucose >200 mg/dL) and nondiabetic mice were subjected to separate groups (*n*=4) and treated as previous. After 20 d, all mice were subjected to 12 h fasting and a single-dose (2.5 g/kg) of glucose was orally gavaged. Blood glucose was measured from tail vein at 0, 30, 60, 120, and 180 min after glucose administration.

### 2.9. HPLC Analysis

In order to be analysed in reverse-phase HPLC and identify relatively polar phenolic compounds, NOSE and NORE were delipified following the method of Bligh and Dyer [14]. In brief, the methanolic extracts were mixed with 4 volumes of chilled acetone prior to incubating at −20°C for 1 h. After incubation, the mixtures were spun at 16,000 × g (4°C for 15 min). The supernatant methanolic fractions were carefully collected and were subjected to thin layer chromatography (TLC) on silica gel plate. Acetic acid (10%) in chloroform was used as solvent, and corresponding bands of secondary metabolites were eluted by acetonitrile after detection with 20% (w/v) Na_2_CO_3_ and diluted Folin–Ciocaltaeu reagent (1 : 3; v/v). Samples were analyzed (20 *μ*L injection; 0.4 mL/mL flow) in a HPLC (Agilent, USA) coupled with Zorbax SB-C18 column (4.6 × 150 mm, 3.5 *μ*m) and equipped with a diode array detector. Gradient concentrations of mobile phase A—methanol (M) and B—water (W) with 0.02% H_3_PO_4_ were 25% A + 75% B for 5 min, 30% A + 70% for 10 min, 45% A + 55% for 30 min, and 80% A + 20% B for 45 min. Peaks were identified by relative retention time (RRT) against respective standards (Sigma, USA; ChromaDex, USA) and spectral patterns. Quantifications were done by postcalibration with response factor of standards.

### 2.10. Molecular Docking Studies

Molecular docking was performed as we describe previously [15]. In brief, X-ray diffraction structures of Nrf2 (nuclear factor erythroid 2-related factor 2; PDB ID: 5FNQ) was obtained from the Research Collaboratory for Structural Bioinformatics (RSCB; http://rcsb.org) Protein Data Bank (PDB) database. DNA counterpart was removed from 2UZK using Autodock Vina V1.5.6. Protein sequence was prepared for docking after removal of water molecules, followed by addition of polar hydrogen atoms. Grid measurement was performed after Gasteiger charge calculation and subsequently the .pdb files were converted into .pdbqt files via SMILES server (https://cactus.nci.nih.gov/translate/). Three-dimensional structures of the compounds were retrieved from the NCBI-PUBCHEM database (https://pubchem.ncbi.nlm.nih.gov), prepared for docking analysis using Autodock Vina. The docked conformations were visualized using PyMol V1.7.4.

### 2.11. Statistical Analysis

Statistical analysis was performed using KyPlot version 5.0 (KyensLab Inc.). Group differences were measured using one-way analysis of variance (ANOVA) followed by Dunnett's post hoc test. Data of OGTT were analyzed using one-way ANOVA. All data are reported as the mean ± SD of six measurements. Linear bivariate correlation (one-tailed) was performed to explore the pairwise association between individual variables by Pearson's method (*r*_P_). *P* < 0.05 was considered significant. Principal component analysis (PCA) was performed to represent and the divergence of systemic and metabolic status of individual mouse from each group (*n*=6) using a correlation-based matrix and represented as a PCA loading plot. Further, the divergence of systemic and metabolic status of individual animals were measured using Pearson's distance with complete linkage method and represented by a dendrogram. Multivariate analysis was performed by Minitab® 18.

## 3. Results and Discussion

The present finding demonstrates that oleander stem and root extract significantly lowers hyperglycaemia and insulin resistance without affecting insulin sensitivity. T1D associated systemic oxidative stress was lowered by improvement of intrinsic antioxidant defence associated with limiting lipid peroxidation. Attenuation of diabetes associated hyperlipidemia, hepatic injury, and nephrotoxicity remained linked with improved antioxidant status. Further, strong molecular interaction of HPLC-identified phytochemicals with Nrf2 indicates oleander mediated antidiabetic protection through antioxidant activities.

**Figure 1:**
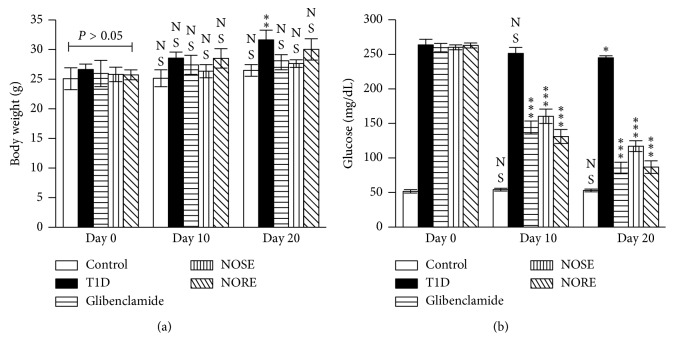
Effects of oleander extracts on body weight (BW) and glucose metabolism. Alloxanized T1D mice (glucose >200 mg/dL) were treated with 200 mg/kg oleander stem (NOSE) and root (NORE) extracts for 20 consecutive days, and glibenclamide (Glib) was used as standard. After the intervention, (a) BW increase of NOSE- and NORE-treated mice were lower compared to day 0, which was otherwise significantly higher in T1D group at day 20. (b) In agreement to the hypothesized antidiabetic activity, blood glucose level was decreased in the alloxanized mice due to NOSE and NORE treatment. Data were represented as mean ± SD of six measurements. ^*∗∗∗*^ *P* < 0.001, ^*∗∗*^ *P* < 0.01, ^*∗*^ *P* < 0.05, and ^NS^ *P* > 0.05 compared to control.

**Figure 2:**
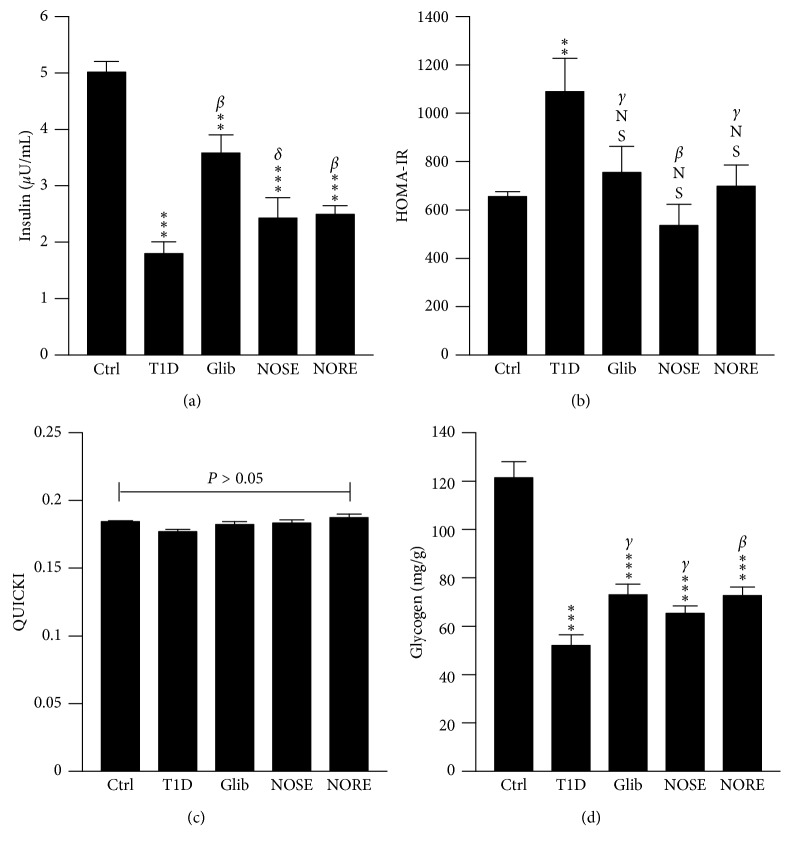
Effects of oleander extracts on the systemic glucose metabolism. The 20 d treatment with NOSE and NORE resulted in the improvement in glucose homeostasis which was otherwise impaired in the alloxanized animals. (a) NOSE and NORE normalized serum insulin level which was associated with systemic glucose load. (b) The elevated insulin resistance, measured by Homeostatic Model Assessment of Insulin Resistance (HOMA-IR) model, was mitigated; however, (c) insulin sensitivity remained unchanged (*P* > 0.05) throughout the treatments as measured by the Quantitative Insulin Sensitivity Check Index (QUICKI) model. (d) Hepatic glycogen level was improved in comparison to T1D group, however not up to the level of control. Data were represented as mean ± SD of six measurements;^*∗∗∗*^ *P* < 0.001, ^*∗∗*^ *P* < 0.01, and ^NS^ *P* > 0.05 compared to control. ^*β*^ *P* < 0.01, ^*γ*^ *P* < 0.05, and ^*δ*^ *P* > 0.05 compared to T1D.

After 20 days of intervention, significant (*P* < 0.01; 19%) BW increase was seen only in case of T1D group (Figure 1(a)). The lowest BW increase was noted (3.15%) for NOSE-treated mice. However, the lowest rate of BW gain was seen in the control (70 mg/d) group. Uncontrolled hyperglycaemia is the hallmark of T1D, and oleander extracts showed profound effects on glucose metabolism in the alloxanized mice. A gradual decrease of blood glucose load was observed in the NOSE and NORE groups (Figure 1(b)). After the intervention, significant (*P* < 0.001) reduction (NOSE: 55% and NORE: 67%) of blood glucose was observed which was otherwise reduced merely 7% in T1D group. Autoimmune pancreatic *β*-cell toxicity lowers circulatory insulin level which leads to persistent hyperglycaemia and abnormal energy metabolism in T1D. However, the etiology of alloxan toxicity leading to insulin-dependent diabetes T1D is governed by *β*-cell oxidative stress and redox imbalance [16]. This was reflected by significantly lower (*P* < 0.001; 64%) serum insulin level in alloxanized mice compared to control (Figure 2(a)). NOSE and NORE considerably increased (*P* < 0.01) the serum insulin level 35% and 32%. Indeed, lowered blood glucose level remained correlated with the increased insulin level (*r*_P_ = −0.847, *P* < 0.001; *r*_P_ = −0772, *P* < 0.01), indicating better glucose utilization by the liver due to improved insulin level. NOSE and NORE further improved insulin resistance which would otherwise remain impaired in the alloxanized mice. In support, HOMA-IR model showed 66% elevated (*P* < 0.001) insulin resistance in T1D mice, indicating alloxan mediated double-diabetes [17], which was 51% and 36% lowered by NOSE and NORE, respectively (Figure 2(b)). In fact, linear association of lowered HOMA-IR with lowered glucose (*r*_P_ = 0.878 to 0.860, *P* < 0.001), and increased insulin (*r*_P_ = −0.585 and −0.412; *P* < 0.05) level highlights improvement of impaired glucose metabolism. This indicates better peripheral uptake of elevated systemic glucose by insulin-target organ and tissue (i.e. skeletal muscle, liver, and adipose) through lowered insulin resistance. However, insulin sensitivity as measured by QUICKI model was marginally impaired (4%; *P* > 0.05) in the alloxanized mice, and compared to T1D group, both the oleander extracts (4–6%) resulted no significant (*P* > 0.05) increase in insulin sensitivity (Figure 2(c)). This suggests that oleander stem and root extracts did not directly attenuate alloxan-induced pancreatic *β*-cell toxicity. Thus, the improved insulin level mediated glucose uptake was likely not due to cytotoxic protection of oleander but due to improved pancreatic insulin signalling and/or improved hepatic glucose metabolism. However, our result contradicts a prior report which concludes hypoglycaemia associated improved insulin sensitivity by oleander shoot distillate in streptozotocin-induced diabetic rats [18]. Thus, future interventions are required to study the effect of oleander on insulin signalling.

Circulatory glucose in excess is converted and stored primarily in the liver as glycogen. Elevated serum glucose level in the alloxanized mice and subsequent increased insulin resistance significantly (*P* < 0.001) decreased the hepatic glycogen level up to 57.14%, which was further improved 26% (*P* > 0.05) and 40% (*P* < 0.01) by NOSE and NORE treatment (Figure 2(d)). Even though, 20 days of intervention failed to restore hepatic glycogen level to control, the improved glycogen level remained associated with improved insulin resistance (*r*_P_ = −0.581 and −0.49; *P* < 0.05) and serum insulin level (*r*_P_ = 0.945 and 0.931; *P* < 0.01), indicating elevated systemic insulin affecting hepatic glycogenesis. Indeed, this was further supported by significant correlation (*r*_P_ = −0.767 and −0.745; *P* < 0.01) between lowered blood glucose and hepatic glycogen level, indicating improved glucose to glycogen turnover in the liver.

**Figure 3:**
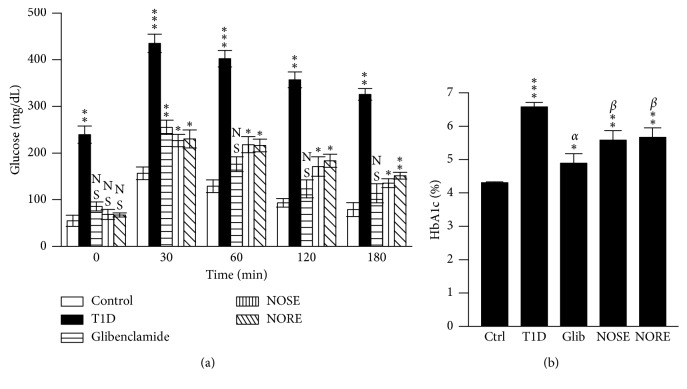
Effects of oleander on oral glucose tolerance (OGTT) and glycated haemoglobin (HbA1c) levels. (a) The treatment effects on acute glucose administration was measured in a separate cohort of diabetic (glucose >200 mg/dL) and nondiabetic control mice, which demonstrated a time-dependent improved peripheral glucose uptake after 20 d of treatment. (b) Improved glycated haemoglobin level further indicated increased glucose metabolism due to 20 d of oleander treatment. Data represented as mean ± SD of 4 measurements for OGTT and 6 measurements for HbA1c. ^*∗∗∗*^ *P* < 0.001, ^*∗∗*^ *P* < 0.01, and ^NS^ *P* > 0.05 compared to control. ^*α*^ *P* < 0.001 and ^*β*^ *P* < 0.01compared to T1D.

The potentials of oleander extracts to improve body's ability to utilize excess glucose was further evaluated by OGTT. Thirty-minute postglucose (2.5 g/kg) gavage, blood glucose was significantly (*P* < 0.001) spiked up in all mice (Figure 3(a)). At postprandial 180 min, the blood glucose level was 25%, 40%, and 34% lower in T1D, NOSE, and NORE groups, respectively, indicating more efficient removal of excess glucose from the circulation and improvement of peripheral glucose uptake due to oleander extracts. In fact, NOSE-treated (*r*_P_ = −0.9942; *P* < 0.01) and NORE-treated (*r*_P_ = −0.9997; *P* < 0.001) groups not only demonstrated comparatively more time-dependent activity than T1D (*r*_P_ = −0.9901; *P* < 0.01), but the rate of initial blood glucose spike was lower in NOSE (Δ_glu/min_: 5.27 mg/dL/min; 231%) and NORE (Δ_glu/min_: 5.43 mg/dL/min; 242%) compared to both T1D (Δ_glu/min_: 6.52 mg/dL/min) and Glib (Δ_glu/min_: 5.64 mg/dL/min). Following postintervention glucose levels, improvement of systemic glucose level was additionally reflected by HbA1c levels (Figure 3(b)). NOSE and NORE significantly (*P* < 0.01) normalized HbA1c level 14–15%, respectively, which was otherwise 53% higher in T1D group compared to control (*P* < 0.001). Lowered HbA1c was in association with lowered blood glucose level (*r*_P_ = 0.940 and 0.866; *P* < 0.01). Moreover, lowered HbA1c remained negatively correlated with serum insulin (*r*_P_ = −0.900 and −0.939; *P* < 0.01) and hepatic glycogen (*r*_P_ = −0.859 and −0.860; *P* < 0.01), highlighting oleander stimulated excess glucose uptake and improvement of glucose metabolism in the alloxanized mice.

The Liver plays a critical role in energy homeostasis by regulating glycolysis, gluconeogenesis, and glycogenesis [19]. Strong evidence indicates interplay between hepatic complications and diabetes [20], which was further reflected by 178%, 100%, 107%, and 40% increase in ACP, ALP, ALT, and AST in the T1D mice (Table 1). All the hepatic injury marker enzymes were lowered due to NOSE and NORE treatment, reflecting improvement of liver injury in the alloxanized T1D mice. This corroborates previous *in vivo* and *in vitro* report showing oleander stem and leaf hydromethanolic extract attenuates haloalkane-induced hepatotoxicity [9]. In support, the extent of lowered hepatic marker enzymes remained in accordance with improved hepatic glycogen (*r*_P_ = −0.938 to −0.692; *P* < 0.01 to 0.001), serum insulin (*r*_P_ = −0.932 to −0.751; *P* < 0.01), and glucose (*r*_P_ = 0.976 to 0.878; *P* < 0.01), demonstrating association of oleander mediated lower hyperglycaemic complications with lowered hepatic injury.

Preclinical and clinical evidence suggest direct association of diabetic hyperlipidaemia with progressive fatty-liver disease [21]. This was evident by significant (*P* < 0.05) 54.88% increase in total serum cholesterol and 51% in triglyceride levels (Table 1). Oleander extracts lowered serum total cholesterol (13% and *P* < 0.05; 29% and *P* < 0.001) and triglyceride (12% and 22%; *P* < 0.05) levels, indicating its hypolipidemic potentials. In support, previous reports have demonstrated that oleander floral extract limits high-fat diet induced dyslipidaemia associated with BW gain [22]. Insulin signalling is known to modulate adiposity and hepatic triglyceride deposition, leading to fatty-liver phenotype in diabetes and obesity [23]. Consequently, improvement of hyperlipidaemia by NOSE and NORE treatment likely resulted through improved insulin resistance. A positive association between lowered triglyceride and insulin resistance (*r*_P_ = 0.654, *P* < 0.05; *r*_P_ = 0.66, *P* < 0.01) further supports this view.

Diabetic nephropathy is the secondary effect of uncontrolled hyperlipidemia, impaired glycaemic homeostasis, and metabolic dysfunction, and alloxan treatment results in interstitial nephritis and tubular atrophy [24]. Acute renal injury in the alloxanized mice was noted by elevation of creatinine (83%, *P* < 0.05), BUN (191%, *P* < 0.05), and uric acid (41%, *P* > 0.05), which serves as markers of nephrotoxicity (Table 1). After oleander treatment, the lowered nephrotoxicity markers remained correlated with lowered serum glucose and triglyceride levels, indicative of limiting hyperglycaemia and hyperlipidemia mediated lowering of diabetic nephropathy [25]. NOSE and NORE treatment lowered serum creatinine, BUN, and uric acid 18 to 24%, 31 to 43, and 16 to 26%, respectively.

**Figure 4:**
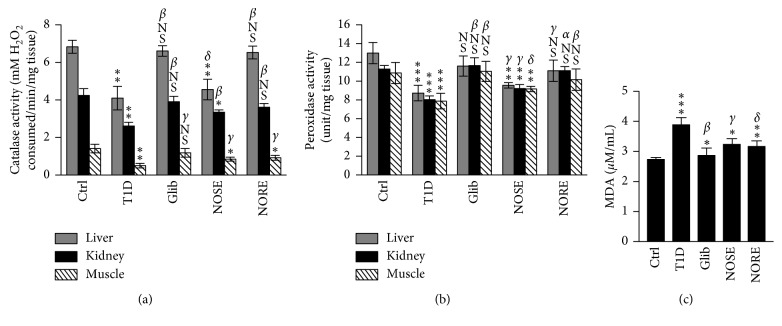
Effects of oleander extracts on intracellular antioxidant protection and systemic oxidative stress in T1D. Consecutive 20 days of treatment of alloxanized mice (glucose >200 mg/dL) with oleander stem (NOSE) and root (NORE) extracts increased the (a) catalase and (b) peroxidase activities in the primary hyperglycaemia-associated tissue of the liver, kidneys, and skeletal muscle. (c) The improved systemic antioxidant defences and lowered intracellular oxidative stress were highlighted by lowered lipid peroxidation (MDA). ^*∗∗∗*^ *P* < 0.001, ^*∗∗*^ *P* < 0.01, ^*∗*^ *P* < 0.05, and ^NS^ *P* > 0.05 compared to control. ^*α*^ *P* < 0.001, ^*β*^ *P* < 0.01, ^*γ*^ *P* < 0.05, and ^*δ*^ *P* > 0.05 compared to T1D.

Hyperglycaemia-related increased glucose-oxidation, cellular protein nonenzymatic glycation, and subsequent degradation results in free-radical generation and redox imbalance [26]. Consequently, progressive glycation of cellular cytoprotective enzymes leads to lowered antioxidative protection and consequently hypersusceptibility to the elevated oxidative stress [27]. Oleander extracts overall improved the systemic antioxidant status in the liver, kidneys, and skeletal muscle as reflected by elevated CAT and PX activities, which were otherwise 27–64% lowered (*P* < 0.001 to 0.01) in alloxanized mice (Figures 4(a) and 4(b)). Liver CAT and muscle PX activities were elevated 11.14% and 16.29% NOSE, irrespective of significant treatment effect compared to T1D group. Improvement of systemic antioxidant enzymatic activities were negatively associated with improved hypoglycaemia (*r*_P_ = −0.927 to −0.822; *P* < 0.01), insulin resistance (*r*_P_ = −0.840 to −0.544; *P* < 0.01 to 0.05), and hepatic ALT level (*r*_P_ = −0.309 to −0.810; *P* < 0.01), which indicates strong association of oleander-mediated improved antioxidative protection with mitigation of hypoglycaemia hepatic injury. This corroborates with previous report demonstrating significant neutralization of the major physiologically relevant intracellular O_2_ and N_2_ free-radicals by hydromethanolic extracts of oleander [7]. The improvement of systemic antioxidant status was further confirmed by measuring serum MDA levels. NOSE and NORE treatments lowered the MDA level 17% (*P* < 0.05) and 18% (*P* < 0.01), respectively, which was or else 42% (*P* < 0.001) higher in T1D compared to control (Figure 4(c)). High association of lowered MDA level with increase CAT and PX activities further supports oleander-mediated improved systemic antioxidant status.

**Figure 5:**
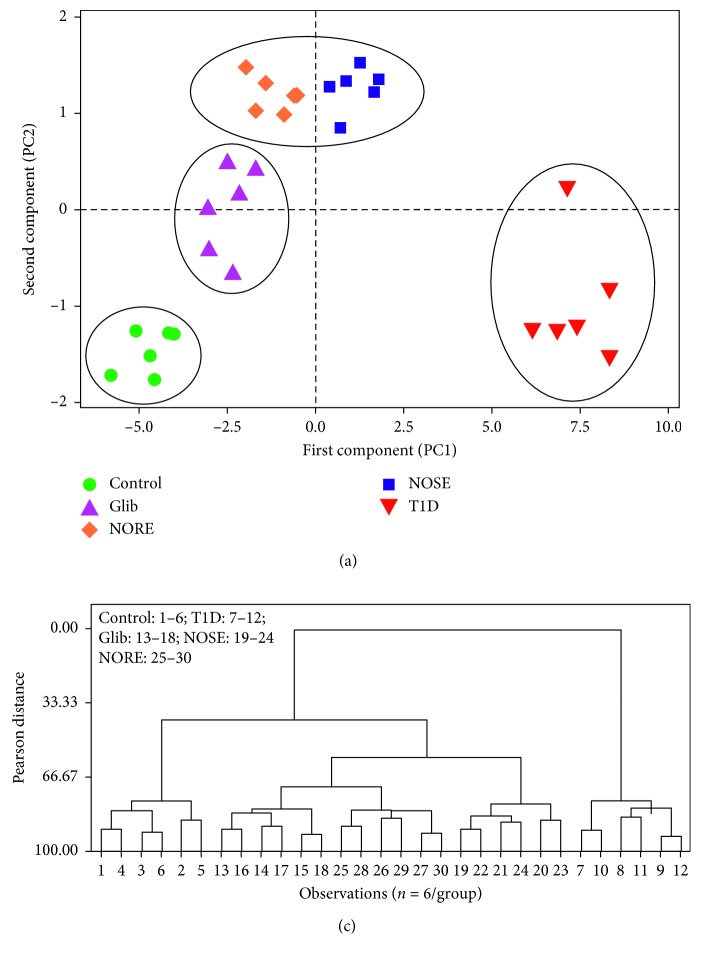
Oleander treatment for 20 consecutive days improves systemic metabolic homeostasis and antioxidant status. (a) The loading plot of principal component analysis (PCA) demonstrated a drastic shift of the spatial arrangement of T1D group mice from the remaining clusters. This was brought back to normal by glibenclamide and oleander treatment, spatial arrangement of which was near to control and far from T1D group. PC1 and PC2 accounted for cumulatively 88.2% and 81.8% variance, respectively. (b) The divergence of 5 different clusters of the 5 experimental groups were measured (Supplementary Materials) and represented using a dendrogram.

Further, multivariate analysis was performed to reflect the systemic shift of metabolic and antioxidative status due to T1D and its mitigation by oleander and standard glibenclamide. In agreement to the systemic shift in the metabolic and antioxidant status, T1D mice were clustered away from the control mice (Figure 5(a)). However, treatment with Glib and oleander extracts in T1D mice resulted in a shift away from T1D and closer to control. This reflected mitigation of T1D complications by the oleander extracts. NOSE and NORE clustered in close proximity; however, NORE-treated mice remained comparatively closer to Glib group and control groups, indicating superior bioactivities of NORE compared to NOSE. Moreover, the nearness of treatment effects on individual animals were measured using Pearson distance based on similarity approach and represented using a dendrogram (Figure 5(b)). This as well confirmed the clustering patterns from PCA loading plot, demonstrating distance divergence of T1D animals (7–12) from the remaining clusters at the root. The control animals (1–6) diverge at Pearson distance 41.02, generating separate branching from the treatment groups. Further at Pearson distance 59.98, NOSE group (19–24) diverge from NOSE and Glib. This reflects the clustering patterns of PCA plot, which indicates nearness of NORE to control and glib compared to NORE. A detailed account of amalgamation steps and final partitions are provided in the Supplementary Materials.

**Figure 6:**
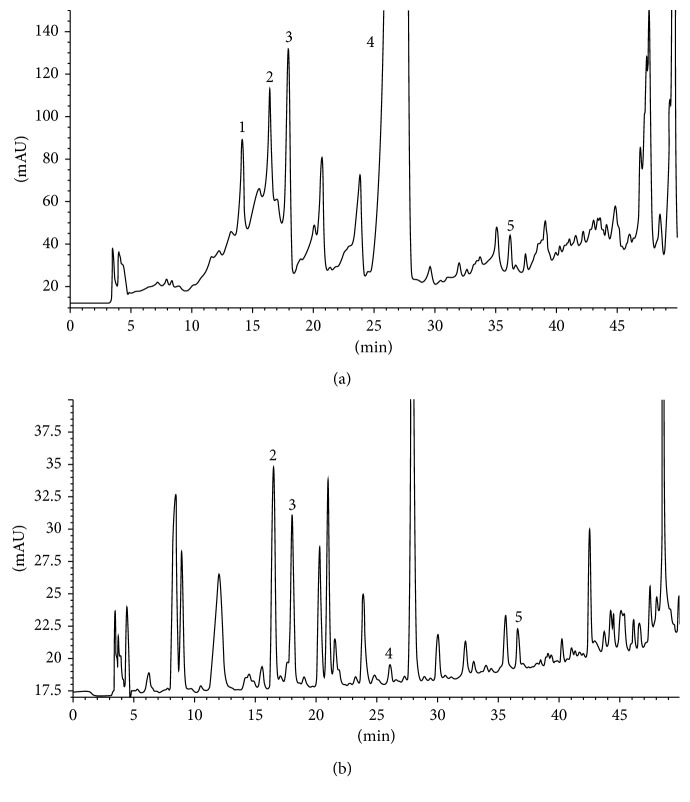
HPLC chromatograms of NOSE and NORE. Measurements were taken at 275 nm. (a) Chromatogram of NOSE and (b) chromatogram of NORE. Peak represents (1) 4-hydroxybenzoic acid; (2) vanillic acid; (3) syringic acid; (4) ferulic acid; (5) myricetin. Chromatogram of reference standards is represented in Supplementary Figure 1.

In the present study (Figures 6(a) and 6(b)), HPLC-UV (275 nm) analysis identified 4-hydroxybenzoic acid (0.321 *μ*g/mg dw), vanillic acid (0.307 *μ*g/mg dw), syringic acid (0.556 *μ*g/mg dw), ferulic acid (2.72 *μ*g/mg dw), and myricetin (0.012 *μ*g/mg dw) in NOSE. Similarly, vanillic acid (0.014 *μ*g/mg dw), syringic acid (0.023 *μ*g/mg dw), ferulic acid (0.0003 *μ*g/mg dw), and myricetin (0.0006 *μ*g/mg dw) were the phenolic compounds identified in NORE. Prior reports suggest that 4-hydroxybenzoic acid exerts antihyperglycaemia by improving peripheral glucose uptake without affecting insulin secretion and hepatic glycogen content [28]. Vanillic acid lowers insulin resistance and dyslipidaemia and glucose uptake in high-fat diet-fed rats [29]. Syringic acid is known to lower hyperglycaemic status in alloxanized mice by elevating plasma insulin and C-peptide levels in systemic circulation [30]. Potent antioxidant and anti-inflammatory activities of ferulic acid have been demonstrated to contributing towards lowering diabetic nephropathy [31]. Moreover, several studies have reported beneficial role of myricetin in mitigating diabetes-related complications such as hyperglycaemia, insulin resistance, hyperlipidemia, obesity, etc. [32]. Apart from the phenolic acids identified in the present study, recent studies have identified a vast array of bioactive constituents in the stem and root of oleander, several of which are well established for their potent antidiabetic activities [8, 9].

**Figure 7:**
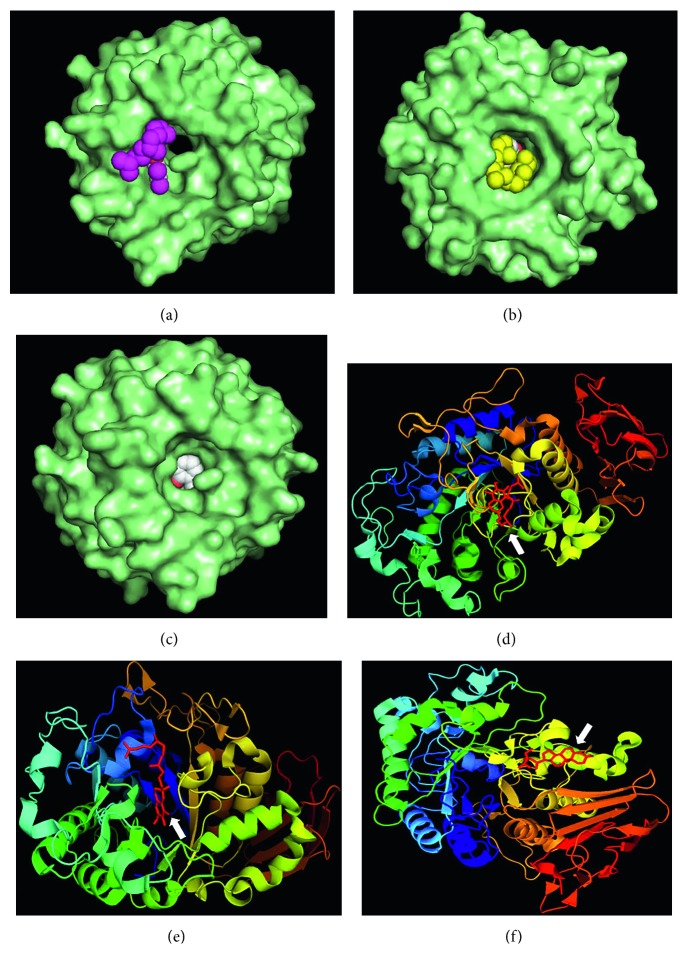
Molecular interaction modelling of HPLC-identified compounds with cytoprotective transcription factor Nrf2. (a, d) 4-hydroxybenzoic acid; (b, e) ferulic acid; (c, f) syringic acid.

Following the trail of oleander mediated improvement of systemic antioxidant status, synergistic effects of the phytochemicals with the redox sensitive transcription factor Nrf2 which regulate intrinsic antioxidant enzymes were studied. A comprehensive molecular docking study of all the phytochemicals being out of scope of the present study, the HPLC-identified phenolic acids were only focused for direct interaction with Nrf2, which transcriptionally regulates the expression of intracellular antioxidant enzymes and its activation is associated with limiting diabetes [33]. Out of the 5 compounds, only ferulic acid, 4-hydroxybenzoic acid, and syringic acid interacted with Nrf2 with high binding energies of −5.2, −5.3, and −5.1 kcal/mol, predicting potential modulation of Nrf2 activation status (Figure 7). Indeed, pharmacological studies also demonstrated that bioactive fractions of oleander induce Nrf2 activation [34].

## 4. Conclusion

The phenotypic observations of the present study therefore provided a convincing evidence that support the use of oleander extracts as antidiabetic remedy in traditional medicine. NOSE and NORE not only limit acute hyperglycaemia and insulin resistance but also attenuates diabetes associated complications such as dyslipidaemia and hepatic and renal injury. Although the molecular mechanism contributing to these pharmacological effects is out of the scope of the present study, indeed data implicate that oleander-induced improvement of systemic antioxidant status is likely responsible for the potent antiiabetic activity. These data collectively indicate the need of further pharmacognostic investigations to identify the additive benefits of the bioactive phytochemicals in oleander.

## Figures and Tables

**Table 1:** Effect of NOSE and NORE on serum enzymatic and biochemical parameters.

	Control	Alloxan			
		T1D	Glibenclamide	NOSE	NORE
ACP	3.39 ± 0.09	8.06 ± 0.76^*∗∗*^	5.79 ± 0.25^*∗∗*^^*δ*^	6.15 ± 0.38^*∗∗*^^*γ*^	5.06 ± 0.24^*∗∗*^^*γ*^
ALP	10.90 ± 1.07	21.85 ± 1.52^*∗*^	11.61 ± 1.25^NS*α*^	14.0 ± 0.37^*∗*^^*γ*^	12.44 ± 0.36^NS*β*^
ALT	43.12 ± 1.57	89.13 ± 7.05^*∗∗*^	54.72 ± 2.30^*∗∗*^^*γ*^	65.27 ± 2.38^*∗∗∗*^^*γ*^	54.88 ± 5.97^NS*γ*^
AST	68.32 ± 8.08	95.87 ± 4.86^NS^	74.90 ± 1.91^NS*γ*^	85.72 ± 3.89^NS*β*^	73.95 ± 2.24^NS*γ*^
Cholesterol	78.31 ± 6.49	121.29 ± 3.04^*∗*^	75.67 ± 3.07^NS*β*^	104.92 ± 2.49^NS*γ*^	85.50 ± 4.44^NS*α*^
Triglyceride	92.1 ± 4.15	139.19 ± 5.77^*∗*^	95.91 ± 4.24^NS*β*^	121.95 ± 5.96^*∗*^^*γ*^	109.97 ± 4.25^*∗*^^*γ*^
Creatinine	0.18 ± 0.01	0.33 ± 0.01^*∗*^	0.19 ± 0.01^NS*γ*^	0.27 ± 0.00^*∗∗*^^*γ*^	0.25 ± 0.01^*∗*^^*β*^
BUN	8.55 ± 1.08	24.90 ± 3.00^*∗*^	12.19 ± 3.08^NS*δ*^	17.20 ± 0.50^*∗*^^*γ*^	14.08 ± 1.02^*∗*^^*γ*^
Uric acid	1.77 ± 0.15	2.50 ± 0.20^NS^	1.90 ± 0.12^NS*α*^	2.09 ± 0.03^NS*δ*^	1.86 ± 0.09^NS*γ*^

^NS^ *P* = nonsignificant (*P* > 0.05), ^*∗*^ *P* < 0.05, ^*∗∗*^ *P* < 0.01, and ^*∗∗∗*^ *P* < 0.001 vs control. ^*α*^ *P* < 0.001, ^*β*^ *P* < 0.01, ^*γ*^ *P* < 0.05, and ^*δ*^ *P* > 0.05 compared to T1D. ACP: acid phosphatase; ALP: alanine aminotransferase; ALT: alanine transaminase; AST: aspartate transaminase; BUN: blood urea nitrogen; NOSE: *Nerium oleander* stem extract; NORE: *Nerium oleander* root extract; T1D: type 1 diabetes.

## Data Availability

The data used to support the findings of this study are available from the corresponding author upon request.

## Supplementary Materials

Supplementary Materials Supplementary Figure 1: HPLC chromatogram of reference standards of phenolic acids and flavonoids. 1: 4-hydroxy benzoic acid (RT: 14.044), 2: vanillic acid (RT: 16.327), 3: syringic acid (RT: 17.873), 4: *p*-coumaric acid (RT: 23.767), 5: ferulic acid (RT: 25.705), 6: rutin (RT: 32.489), 7: myricetin (RT: 36.144), and 8: quercetin (42.217).

## References

[1] WHO (2016). *Diabetes*.

[2] IDF (2016). *IDF Diabetes Atlas*.

[3] Dey P., Chaudhuri T. K. (2014). Pharmacological aspects of Nerium indicum Mill: a comprehensive review. *Pharmacognosy Reviews*.

[4] Mwafy S. N., Yassin M. M. (2011). Antidiabetic activity evaluation of glimepiride and *Nerium oleander* extract on insulin, glucose levels and some liver enzymes activities in experimental diabetic rat model. *Pakistan Journal of Biological Sciences*.

[5] Dey P., Saha M. R., Chowdhuri S. R. (2015). Assessment of anti-diabetic activity of an ethnopharmacological plant *Nerium oleander* through alloxan induced diabetes in mice. *Journal of Ethnopharmacology*.

[6] Sikarwar M. S. P. M., Kokate C. K., Sharma S., Bhat V. (2009). Antidiabetic activity of nerium indicum leaf extract in alloxan-induced diabetic rats. *Journal of Young Pharmacists*.

[7] Dey P., Chaudhuri D., Chaudhuri T. K., Mandal N. (2012). Comparative assessment of the antioxidant activity and free radical scavenging potential of different parts of Nerium indicum. *International Journal of Phytomedicine*.

[8] Dey P., Chaudhuri T. K. (2016). Comparative phytochemical profiling and effects of *Nerium oleander* extracts on the activities of murine peritoneal macrophages. *Archives of Biological Sciences*.

[9] Dey P., Dutta S., Biswas-Raha A., Sarkar M. P., Chaudhuri T. K. (2016). Haloalkane induced hepatic insult in murine model: amelioration by Oleander through antioxidant and anti-inflammatory activities, an in vitro and in vivo study. *BMC Complementary and Alternative Medicine*.

[10] Pettersson U. S., Walden T. B., Carlsson P. O., Jansson L., Phillipson M. (2012). Female mice are protected against high-fat diet induced metabolic syndrome and increase the regulatory T cell population in adipose tissue. *PLoS One*.

[11] Carroll N. V., Longley R. W., Roe J. H. (1956). The determination of glycogen in liver and muscle by use of anthrone reagent. *Journal of Biological Chemistry*.

[12] Sadasivam S. (1996). *Biochemical Methods*.

[13] Lück H. (1965). Catalase. *Methods of Enzymatic Analysis*.

[14] Bligh E. G., Dyer W. J. (1959). A rapid method of total lipid extraction and purification. *Canadian Journal of Biochemistry and Physiology*.

[15] Saha M. R., Dey P., Sarkar I. (2018). *Acacia nilotica* leaf improves insulin resistance and hyperglycemia associated acute hepatic injury and nephrotoxicity by improving systemic antioxidant status in diabetic mice. *Journal of Ethnopharmacology*.

[16] Lenzen S. (2007). The mechanisms of alloxan- and streptozotocin-induced diabetes. *Diabetologia*.

[17] Ader M., Richey J. M., Bergman R. N. (1998). Evidence for direct action of alloxan to induce insulin resistance at the cellular level. *Diabetologia*.

[18] Bas A. L., Demirci S., Yazihan N., Uney K., Ermis Kaya E. (2012). *Nerium oleander* distillate improves fat and glucose metabolism in high-fat diet-fed streptozotocin-induced diabetic rats. *International Journal of Endocrinology*.

[19] Dey P., Saha M. R., Sen A. (2013). Hepatotoxicity and the present herbal hepatoprotective scenario. *International Journal of Green Pharmacy*.

[20] Loria P., Lonardo A., Anania F. (2013). Liver and diabetes. A vicious circle. *Hepatology Research*.

[21] Regnell S. E., Lernmark A. (2011). Hepatic steatosis in type 1 diabetes. *Review of Diabetic Studies*.

[22] Gayathri V., Ananthi S., Chandronitha C., Sangeetha M. K., Vasanthi H. R. (2011). Hypolipidemic potential of flowers of *Nerium oleander* in high fat diet-fed Sprague Dawley rats. *Natural Product Research*.

[23] Kawano Y., Cohen D. E. (2013). Mechanisms of hepatic triglyceride accumulation in non-alcoholic fatty liver disease. *Journal of Gastroenterology*.

[24] Evan A. P., Mong S. A., Connors B. A., Aronoff G. R., Luft F. C. (1984). The effect of alloxan, and alloxan-induced diabetes on the kidney. *Anatomical Record*.

[25] Woodrow G., Brownjohn A. M., Turney J. H. (1994). Acute renal failure in patients with type 1 diabetes mellitus. *Postgraduate Medical Journal*.

[26] Newsholme P., Haber E. P., Hirabara S. M. (2007). Diabetes associated cell stress and dysfunction: role of mitochondrial and non-mitochondrial ROS production and activity. *Journal of Physiology*.

[27] McGrowder D. A., Anderson-Jackson L., Crawford T. V. (2013). *Biochemical Evaluation of Oxidative Stress in Type 1 Diabetes*.

[28] Peungvicha P., Thirawarapan S. S., Watanabe H. (2001). Possible mechanism of hypoglycemic effect of 4-hydroxybenzoic acid, a constituent of Pandanus odorus root. *Japanese Journal of Pharmacology*.

[29] Chang W.-C., Wu J., Chen C.-W. (2015). Protective effect of vanillic acid against hyperinsulinemia, hyperglycemia and hyperlipidemia via alleviating hepatic insulin resistance and inflammation in High-Fat Diet (HFD)-fed rats. *Nutrients*.

[30] Muthukumaran J., Srinivasan S., Venkatesan R. S., Ramachandran V., Muruganathan U. (2013). Syringic acid, a novel natural phenolic acid, normalizes hyperglycemia with special reference to glycoprotein components in experimental diabetic rats. *Journal of Acute Disease*.

[31] Choi R., Kim B. H., Naowaboot J. (2011). Effects of ferulic acid on diabetic nephropathy in a rat model of type 2 diabetes. *Experimental and Molecular Medicine*.

[32] Li Y., Ding Y. (2012). Minireview: therapeutic potential of myricetin in diabetes mellitus. *Food Science and Human Wellness*.

[33] Uruno A., Furusawa Y., Yagishita Y. (2013). The Keap1-Nrf2 system prevents onset of diabetes mellitus. *Molecular and Cellular Biology*.

[34] Van Kanegan M. J., Dunn D. E., Kaltenbach L. S. (2016). Dual activities of the anti-cancer drug candidate PBI-05204 provide neuroprotection in brain slice models for neurodegenerative diseases and stroke. *Scientific Reports*.

