# Supplementary material for: Oleander Stem and Root Standardized Extracts Mitigate Acute Hyperglycaemia by Limiting Systemic Oxidative Stress Response in Diabetic Mice
Source: Adv Pharmacol Sci. 2019 Jan 8;2019:7865359. doi: 10.1155/2019/7865359 (PMC6341266; doi:10.1155/2019/7865359)
Supplement: Supplementary Materials — Supplementary Figure 1: HPLC chromatogram of reference standards of phenolic acids and flavonoids. 1: 4-hydroxy benzoic acid (RT: 14.044), 2: vanillic acid (RT: 16.327), 3: syringic acid (RT: 17.873), 4: p-coumaric acid (RT: 23.767), 5: ferulic acid (RT: 25.705), 6: rutin (RT: 32.489), 7: myricetin (RT: 36.144), and 8: quercetin (42.217). [file 7865359.f1.pdf]

SUPPLEMENTARY DATA

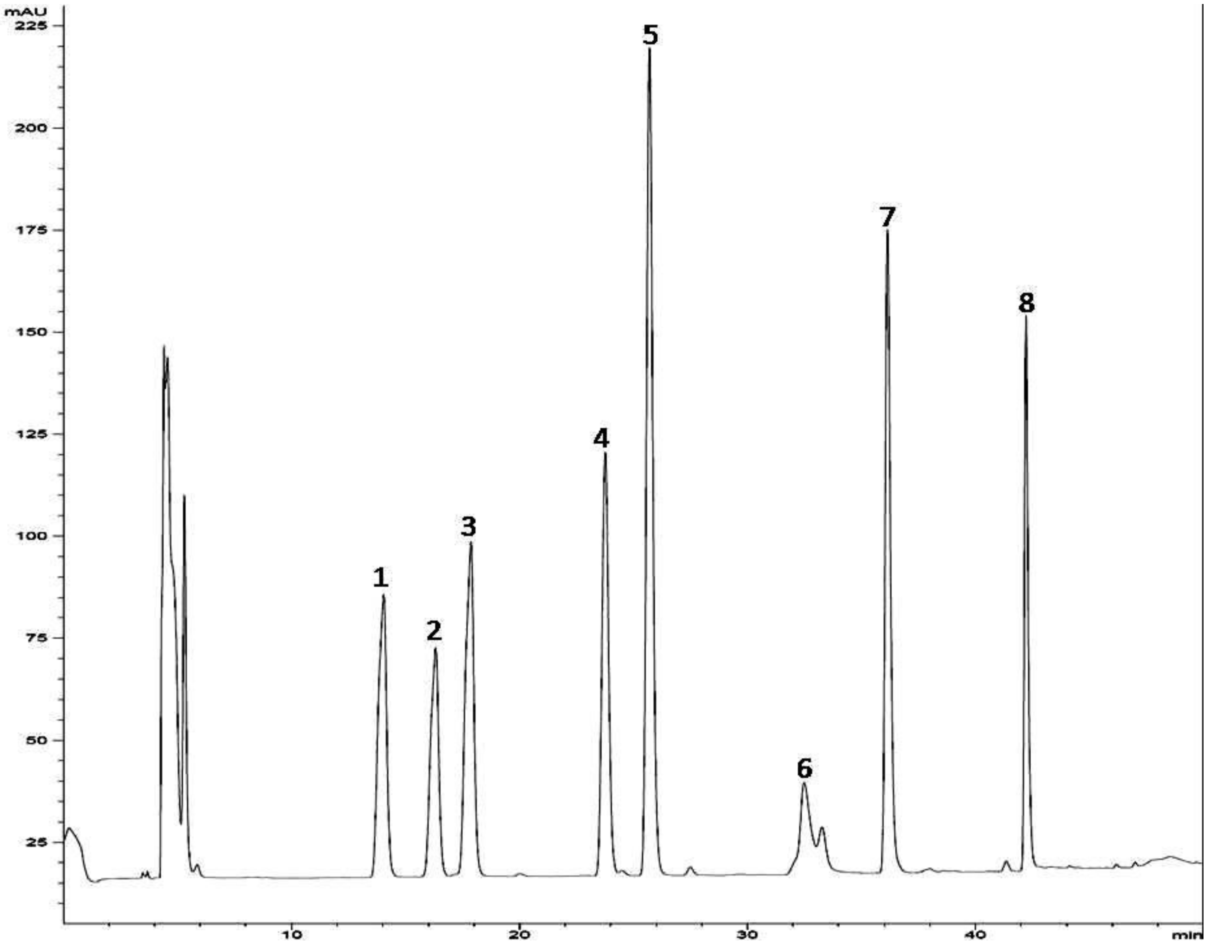

Fig 1. HPLC chromatogram of reference standards of phenolic acids and flavonoids. ***1:*** 4-Hydroxu benzoic acid (RT: 14.044), ***2:*** Vanillic acid (RT: 16.327), ***3:*** Syringic acid (RT: 17.873), ***4:*** p-Coumaric acid (RT: 23.767), ***5:*** Ferulic acid (RT: 25.705), ***6:*** Rutin (RT: 32.489), ***7:*** Myricetin (RT: 36.144), ***8:*** Quercetin (42.217).

Amalgamation Steps

Step	Number of clusters	Similarity level	Distance level	Clusters joined		New cluster	Number of obs. in new cluster
1	29	93.3428	0.9544	9	12	9	2
2	28	92.3068	1.1029	15	18	15	2
3	27	92.2266	1.1144	27	30	27	2
4	26	91.5248	1.2151	3	6	3	2
5	25	90.6388	1.3421	7	10	7	2
6	24	90.1002	1.4193	1	4	1	2
7	23	89.9885	1.4353	13	16	13	2
8	22	89.9830	1.4361	19	22	19	2
9	21	89.0523	1.5695	25	28	25	2
10	20	88.7894	1.6072	14	17	14	2
11	19	87.2350	1.8301	21	24	21	2
12	18	85.9660	2.0120	20	23	20	2
13	17	85.9200	2.0186	2	5	2	2
14	16	85.1536	2.1285	26	29	26	2
15	15	84.8969	2.1653	8	11	8	2
16	14	84.2596	2.2566	19	21	19	4
17	13	83.0872	2.4247	13	14	13	4
18	12	82.1036	2.5657	26	27	26	4
19	11	81.9009	2.5948	1	3	1	4
20	10	81.6460	2.6314	25	26	25	6
21	9	81.3119	2.6793	13	15	13	6
22	8	80.5845	2.7835	8	9	8	4
23	7	78.7545	3.0459	19	20	19	6
24	6	77.5707	3.2156	7	8	7	6
25	5	77.2848	3.2566	1	2	1	6
26	4	71.1151	4.1411	13	25	13	12
27	3	57.9775	6.0246	13	19	13	18
28	2	41.0117	8.4570	1	13	1	24
29	1	0.0000	14.3367	1	7	1	30

Final Partition

	Number of observations	Within cluster sum of squares	Average distance from centroid	Maximum distance from centroid
Cluster1	30	1555583	176.833	492.941
